# Accuracy of the Apple Watch Oxygen Saturation Measurement in Adults: A Systematic Review

**DOI:** 10.7759/cureus.35355

**Published:** 2023-02-23

**Authors:** Paul Windisch, Christina Schröder, Robert Förster, Nikola Cihoric, Daniel R Zwahlen

**Affiliations:** 1 Department of Radiation Oncology, Kantonsspital Winterthur, Winterthur, CHE; 2 Department of Radiation Oncology, Inselspital, University Hospital of Bern, Bern, CHE

**Keywords:** telehealth, wearables, apple watch, pulse oximetry, oxygen saturation, digital health

## Abstract

The purpose of this review is to summarize the research on the accuracy of oxygen saturation (spO_2_) measurements using the Apple Watch (Apple Inc., Cupertino, California). The Medline and Google Scholar databases were searched for papers evaluating the spO_2_ measurements of the Apple Watch vs. any kind of ground truth and records were analyzed according to the Preferred Reporting Items for Systematic Reviews and Meta-Analyses (PRISMA) guidelines.

The five publications with 973 total patients that met the inclusion criteria all used the Apple Watch Series 6 and described 95% limits of agreement of +/- 2.7 to 5.9% spO_2_. However, outliers of up to 15% spO_2_ were reported. Only one study had patient-level data uploaded to a public repository. The Apple Watch Series 6 does not show a strong systematic bias compared to conventional, medical-grade pulse oximeters. However, outliers do occur and should not cause concern in otherwise healthy individuals. The impact of race on measurement accuracy should be investigated.

## Introduction and background

Wearables are blurring the lines between lifestyle devices and medical products. In the case of the Apple Watch (Apple Inc., Cupertino, California), some features, like the electrocardiogram (ECG) function have been evaluated in large studies and received clearance by the United States Food and Drug Administration (FDA) while others have not and are framed as wellness trackers [1].

Despite this distinction, researchers have started investigating the potential use of these non-approved features for remote monitoring settings in the future [2]. An already existing issue is that many users who just use the watch as a lifestyle device are likely unaware of the distinction and might approach their providers with questions regarding conspicuous values measured by the watch. The ability of the watch to provide oxygen saturation (spO_2_) measurements is one of those features that has previously been available only with medical-grade pulse oximeters.

However, unlike medical-grade pulse oximeters, the Apple Watch can't use the common transmissive pulse oximetry where light is passed through a thin part of the body, and instead has to rely on reflectance pulse oximetry where light is passed into the wrist and only the reflected light is measured at the photodiode. This approach is considered more challenging as changes in spO_2_ tend to produce smaller changes in the signals which also appear to be less stable [3].

With this technology now being available to a large population, the purpose of this systematic review is therefore to summarize the research that has been published to date to provide information on the accuracy of the measurements and guide further research.

## Review

The review was conducted according to the Preferred Reporting Items for Systematic Reviews and Meta-Analyses (PRISMA) guidelines [4]. Original articles that evaluated the spO_2_ measurement feature of any Apple Watch series against any kind of ground truth were included. No constraints regarding the language of the publication were applied. Articles had to be published no earlier than 2015.

The Medical Literature Analysis and Retrieval System Online (MEDLINE) database were searched on October 18th, 2022, via the PubMed interface. The query was designed to include studies with either the words "Apple Watch" or "Apple Smartwatch" in the title or abstract as well as at least one word indicating the investigation of oxygen saturation. The complete search query that was used for PubMed was therefore: "(Apple Watch[title/abstract]) OR (Apple Smartwatch[title/abstract]) AND (spO2[title/abstract] OR oxygen[title/abstract] OR oximetry[title/abstract]) AND ("2015/01/01"[Date - Publication]: "2022/10/18"[Date - Publication])".

For Google Scholar, two separate queries were used, and the filtering by year of publication was done through the user interface: 'allintitle: SpO2 OR Oxygen OR Oximetry "Apple SmartWatch"', and 'allintitle: SpO2 OR Oxygen OR Oximetry "Apple Watch"'.

**Figure 1:**
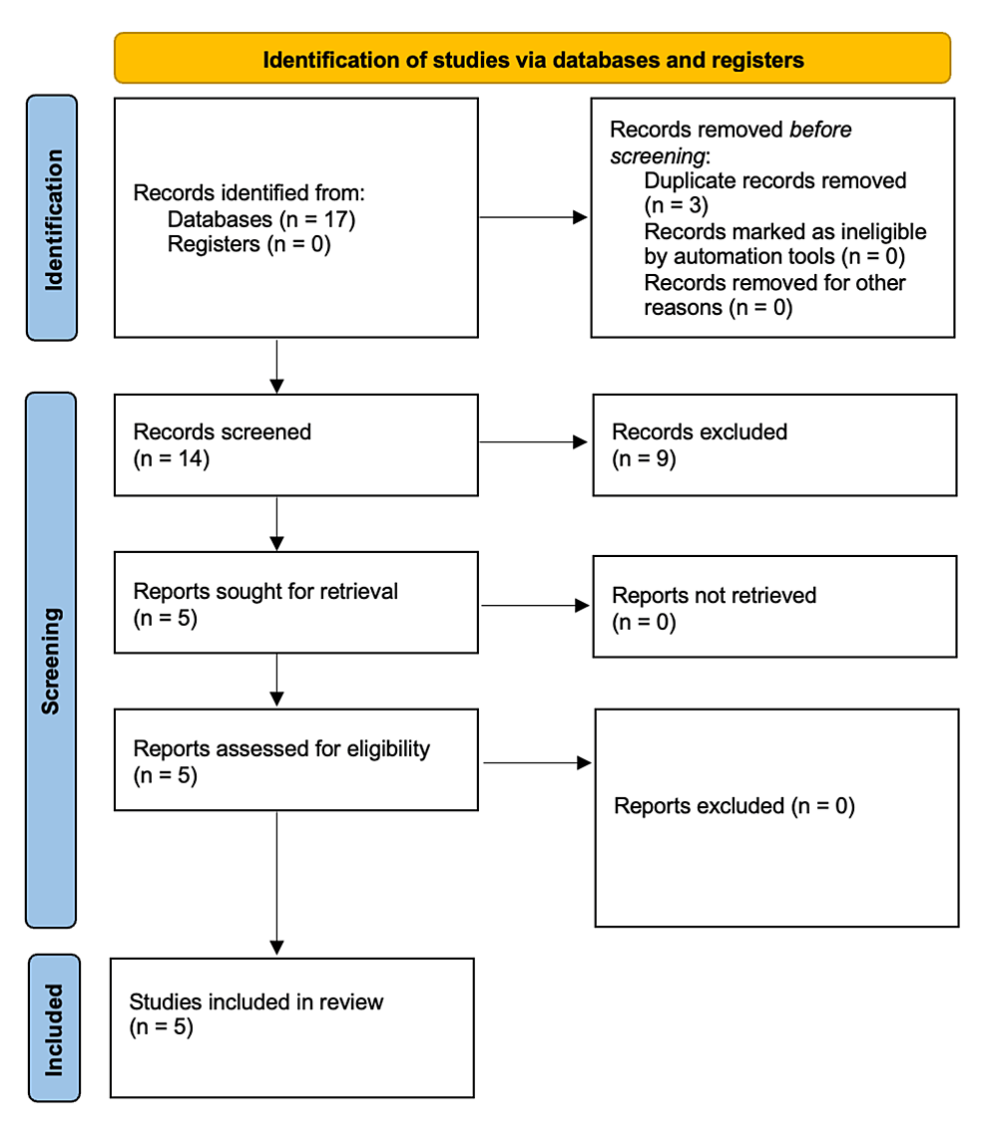
Workflow of the literature search according to PRISMA guidelines Source: Page et al. [4] PRISMA - Preferred Reporting Items for Systematic Reviews and Meta-Analyses

After the exclusion of duplicates, the titles, as well as abstracts, were screened, and only relevant publications proceeded to full-text screening. The decision as to whether a study met the inclusion criteria of the review was made by two authors (PW, CS) without using automated tools. A third author (DRZ) acted as a referee in case of a potential disagreement between the two authors responsible for screening. The review had not been registered beforehand, and no protocol had been published. Two authors (PW and CS) independently extracted data and discussed any discrepancies. Data were extracted with regards to 1) Study parameters (title, authors, year of publication, number of patients); 2) clinical parameters (conditions, patient age, spO2 measurements); 3) technical parameters (Apple Watch series, wristband, ground truth); and 4) study parameters (data availability, conflict of interest, funding). The inclusion workflow is depicted in Figure 1.

The query returned 17 articles (12 from PubMed, and five from Google Scholar), three of which were duplicates. Eight articles were original articles that were excluded due to not comparing Apple Watch spO_2_ measurements to a ground truth [5-12]. One article was excluded due to being an editorial [13]. All excluded articles and the respective reasons for exclusion are provided in Table 1.

**Table 1:** Excluded studies and reason for exclusion

Authors	Title	Year	Reason for exclusion
Yamagami et al. [5]	Early Detection of Symptom Exacerbation in Patients With SARS-CoV-2 Infection Using the Fitbit Charge 3 (DEXTERITY): Pilot Evaluation	2021	Original article but out of scope
O'Neill et al. [6]	2-Hydroxybenzylamine (2-HOBA) to Prevent Early Recurrence of Atrial Fibrillation After Catheter Ablation: Protocol for a Randomized Controlled Trial Including Detection of AF Using a Wearable Device	2021	Original article but out of scope
Chandrasekaran et al. [7]	Patterns of Use and Key Predictors for the Use of Wearable Health Care Devices by US Adults: Insights From a National Survey	2020	Original article but out of scope
LaMunion et al. [8]	Use of Consumer Monitors for Estimating Energy Expenditure in Youth	2020	Original article but out of scope
Wilson [13]	New Apple Watch Monitors Blood Oxygen - Is That Useful?	2020	Not original article (editorial)
Abt et al. [9]	Walking Cadence Required to Elicit Criterion Moderate-Intensity Physical Activity Is Moderated by Fitness Status	2019	Original article but out of scope
Xie et al. [11]	Evaluating the Validity of Current Mainstream Wearable Devices in Fitness Tracking Under Various Physical Activities: Comparative Study	2018	Original article but out of scope
Abt et al. [12]	Measuring Moderate-Intensity Exercise with the Apple Watch: Validation Study	2018	Original article but out of scope
Abt et al. [10]	The Validity and Inter-device Variability of the Apple Watch™ for Measuring Maximal Heart Rate	2018	Original article but out of scope

The remaining five articles were included [2,14-17]. A summary of extracted characteristics is available in Table 2.

**Table 2:** Summary of extracted study parameters

Publication (year)	Device (size)	Wrist band	Ground truth	Number of patients & condition	Age (range) [years]	SpO_2_ according to ground truth (range) [%]	Pearson correlation coefficient	Race	Data availability	Conflict of interest	Funding
Rafl et al. (2022) [17]	Series 6 (not mentioned)	Not mentioned	Masimo Radical-7 pulse oximeter	24 healthy adults in hypoxic conditions	Mean: 24 (20 - 28)	Not mentioned	Not mentioned	Caucasian	Upon request from the corresponding author	None	Czech Technical University
Pätz et al. (2022) [14]	Series 6 (44 mm)	adjustable elastic wristband made of rubber	GE Healthcare Carescape Dinamap V100 pulse oximeter	508 patients (238 adults, 270 children) with congenital heart disease	Adults median: 32 (18 - 76); Children median: 10 (0.1 - 17)	Adults median: 97 (78 - 100); Children median: 97 (73 - 100)	0.813	Not mentioned	Not mentioned	None	Not mentioned
Littell et al. (2022) [16]	Series 6 (not mentioned)	Not mentioned	Coviden Nellcor Portable SpO_2_ Patient Monitoring System	84 patients <23 years with weight >3 kg with an ECG ordered as part of their medical care	Mean: 7.2 (0.1 - 18)	Median: 98 (78 - 100)	0.76	Not mentioned	Uploaded to public repository	None	None
Spaccarotella, Polimeni et al. (2022) [2]	Series 6 (not mentioned)	Not mentioned	Nellcor Portable SpO_2_ Patient Monitoring System, PM10N	257 adult patients (141 with cardiovascular disease, 60 with lung disease, 56 healthy controls)	Mean: 64.0 (not mentioned)	Not mentioned	0.89	White	Upon request from the corresponding author	None	None
Pipek et al. (2021) [15]	Series 6 (44 mm)	small/medium and large	Mobil POD-2 Finger Oximeter, Multilaser OX-06 Oximeter	100 adult patients (23 with COPD, 61 with interstitial lung disease, 16 healthy controls)	Mean: 59.6 (not mentioned)	Mean: 94.4% (not mentioned)	0.81	Assessed with Fitzpatrick skin type, but no information on distribution	Not mentioned	None	Not mentioned

All publications declared that no conflict of interest was present. Two publications declared that no funding for the project had been received [2,16], and one publication declared a grant from the university as a funding source [17], while the remaining two publications did not mention funding [14,15].

Littell and colleagues were the only author group that uploaded data to a public repository [16]. Two of the remaining publications mentioned the data being available from the corresponding author upon reasonable request [2,17], while the others did not mention data availability [14,15].

Pipek et al. evaluated the performance of an Apple Watch Series 6 in 100 adult patients [15]. Measurements were conducted with the Apple Watch worn according to the manufacturer's instructions and one pulse oximeter clip on the index and middle finger of the same hand. All values were noted at the moment the Apple Watch finished its measurement. Of the 100 patients, 61 had a history of interstitial lung disease, 23 had a history of COPD, and 16 were healthy controls. The mean spO_2_ measured with the conventional pulse oximeters was 94.4%. The mean spO_2_ measured with the Apple Watch was 95.9%. The Pearson correlation coefficient between watch and conventional oximeter measurements was 0.81, with limits of agreement of -2.7% and +4.1%. The Pearson correlation coefficient between the two conventional oximeter measurements was 0.993. The authors concluded that the Apple Watch Series 6 was a reliable way to obtain spO_2_ measurements in patients with lung diseases under controlled conditions.

Spaccarotella et al. evaluated the performance of an Apple Watch Series 6 in 257 adult patients [2]. Measurements were conducted according to the manufacturer's instructions on the same arm within one minute of each other. All measurements were performed twice and then averaged. Of the 257 patients, 141 had a history of cardiovascular disease, 60 had a history of lung disease, and 56 were healthy controls. The mean age was 64 years. While the study did not mention the median spO_2_, the Pearson correlation coefficient was 0.89, with limits of agreement of -3.5% and +3.0%. The authors conclude that the watch could be used to assess spO_2_ in healthy patients, as well as in those with cardiovascular or lung disease. 

Littell et al. evaluated the performance of an Apple Watch series 6 in 84 patients with an age of <23 years but weight >3 kg who had an ECG ordered as part of their routine clinical care at a pediatric cardiology unit [16]. For the spO_2_ measurement, patients were instructed to remain still for 15 seconds while the watch was placed on their wrists. In small children <10 kg, the watch was placed on other areas of the body with a bigger surface area, such as the palm of the hand, dorsum of foot, or around calf/thigh if a first measurement on the wrist had failed. The mean age was 7.2 years (range: 0.1 - 18 years). While 37% of patients had no cardiac history, the remaining 63% had either structural heart disease (41%), electrical abnormalities (14%), or both (8%). Twelve patients (14%) had no successful measurement with the Apple Watch even though no formal constraint regarding the number of attempts was applied. One patient had no conventional pulse oximetry data available. Median spO_2_ for the remaining 71 patients, according to the conventional pulse oximeter, was 98% (range: 78 - 100%). The average absolute difference between the Apple Watch and the conventional pulse oximeter was 2.0% spO_2_. The Pearson correlation coefficient between watch and conventional oximeter measurements was 0.76. In four measurements (5%), the watch underestimated the true oxygen saturation by more than 5% spO_2_, and in one case by as much as 15%. While limits of agreement were not mentioned in the text, they are depicted in the paper at around -7 and +5%. The authors concluded that the watch can be used to obtain pulse oximetry in a broad pediatric population.

Pätz et al. evaluated the performance of a 44 mm Apple Watch Series 6 in 508 adults (n=238) and children (n=270) with congenital heart disease [14]. Patients were instructed to sit or lay down in a comfortable position. Three measurements with the Apple Watch were taken while measuring simultaneously with the standard pulse oximeter. If a measurement failed, the watch was moved slightly in between attempts. The median age of the adult population was 32 years (range: 18 - 76 years), and the median age of the children was 10 years (range: 0.1 - 17 years). Median spO_2_ measured with the conventional pulse oximeter was 97% for adults (range: 78 - 100%) as well as children (range: 73 - 100%). The researchers started with measurements where the watch was only laid on the wrist, but after measuring 259, patients started tying the watch properly, which decreased the proportion of unsuccessful measurements after three attempts from 21% to 4%. The authors considered the performance of the watch as correct when the median of the three watch measurements deviated from the standard pulse oximeter measurement by 3% spO_2_ at most, which was the case in 84% of successfully measured adult patients. However, cases where the watch measurements varied by more than 4% spO_2_ were considered unsuccessful measurements and are therefore not included. The Pearson correlation coefficient between the watch and conventional oximeter measurements for cases where the watch was tied around the wrist was 0.813. The authors concluded that due to unsuccessful or incorrect measurements, the Apple Watch is not yet up to the medical standard of conventional pulse oximeters.

Rafl et al. evaluated the performance of an Apple Watch Series 6 in 24 healthy adults that were exposed to hypoxic conditions [17]. The watch measurements were taken from the left wrist according to the manufacturer's instructions with the conventional pulse oximeter being attached to the middle finger of the same hand. Two measurements were taken during a two-minute stabilization phase where participants breathed ambient air. In the following five minutes, the desaturation phase, participants breathed hypoxic air (12% O_2_), and spO_2_ was measured every 30 seconds. In the following stabilization phase, participants breathed ambient air again, and spO_2_ was measured every 30 seconds until it returned to normal values. Each participant underwent the procedure twice, with at least one hour in between, resulting in 642 spO_2_ measurements for the whole study. The mean age of participants was 24 years. The spO_2_ readings in the study range from 76 - 100% though it is unclear on which device this range is based. The authors report 95% limits of agreement of -5.8% and +5.9%. In patients and conditions where the conventional pulse oximeter measured >90% spO_2_, the Apple Watch measured on average 1% higher than the conventional device. The authors concluded that the spO_2_ measuring was sufficiently advanced for indicative measurement outside of the clinic.

While we found several studies that evaluated the spO_2_ measurements of the Apple Watch compared to a conventional, medical-grade pulse oximeter as the ground truth, differences in the testing procedures and the presentation of the resulting data made it difficult to compare between publications and prevented us from attempting a quantitative synthesis.

This applies in particular to the way the watch was worn during testing. The publication by Pätz et al. demonstrates that using an appropriate wristband has an impact on the percentage of failed measurements which also poses the question of how much the accuracy of the measurements is affected [14]. This question also applies to the study by Littell et al., which was the other study that included pediatric patients and the only one that allowed for the watch to be placed on other regions of the body besides the wrist [16]. It should be noted that Apple makes the spO_2_ measurements only available to users who have entered an age >= 18 years [18].

Four of the five studies drew positive conclusions regarding the usability of the spO_2_ measurements in their respective populations. This was mainly based on the fact that no study demonstrated a strong systematic bias of the watch measurements. However, the question if the Apple Watch is appropriate for spO_2_ measurements should always be discussed and viewed in the context of the respective clinical scenario.

The most common scenario today is a supposedly healthy individual using the Apple Watch as a wellness tracker. The only way the watch could provide a benefit in this scenario is by alerting those people who are actually not completely healthy but have an underlying issue that causes a decreased oxygen saturation. The question remains as to whether the watch will be the thing that makes someone see a physician earlier than actual symptoms such as shortness of breath during physical activity.

For the vast majority of individuals using the watch as a wellness tracker, it will likely not provide a meaningful benefit in terms of health guidance and might even cause psychological distress to those who worry about outliers that the watch occasionally produces. Since the normal range for oxygen saturation is 95 - 100%, and the limits of agreement of the reviewed studies are in the +/- 2.7 to 5.9% range, it is to be expected that users will eventually be shown measurements outside of the normal range which, if occurring in isolation, should be no cause for concern.

Whether the watch is suited for remote monitoring in patients with established conditions that affect their oxygen saturation depends on the goal of that monitoring. Since the Apple Watch does not conduct permanent measurements but rather conducts measurements several times during the day, it is not suitable to detect sudden drops in spO_2_ since those might happen outside of measurements. However, if the goal is to see how the oxygen saturation for a patient changes on average, especially over longer periods of time, the watch could be a good and convenient option. 

A question that remains to be addressed is if the accuracy of the measurements depends on skin color, which has also been investigated for conventional pulse oximeters where differences in the risk of occult hypoxemia have been reported [19,20]. Only the study by Pipek and colleagues investigated this question. The other publications either described their populations as homogeneously caucasian (Rafl et al.) or white (Spaccarotella et al.) or didn't cover the subject. While Pipek and colleagues did not find a significant difference in the measurements due to skin color assessed using the Fitzpatrick type, the distribution of skin color among their participants is unclear [15].

Many of the uncertainties mentioned previously could be addressed and mitigated by articles publishing patient-level data like the publication by Littell and colleagues [16]. Ideally, this data would not only contain spO_2_ measurements but also additional information such as Fitzpatrick skin type, skin abnormalities, temperature, wrist circumference, etc.

Possible limitations at the review level include the fact that only articles with "Apple Watch" or "Apple Smartwatch" in their title or abstract were retrieved by the query, which could have led to articles using only terms like "wearable" being missed. However, it seems appropriate to assume that any research using the Apple Watch is likely to use one of the terms somewhere in either title or the abstract. In addition, we also did not notice any papers in the discussion sections of the included manuscripts that were not returned by our query.

Only two databases were queried, but this limitation is mitigated by the fact that the majority of publications in the field of digital health appear in PubMed or Google Scholar-indexed journals. 

Limitations at the study level include the heterogeneous measurement and reporting processes. In addition, all studies used conventional, medical-grade oximeters as the ground truth instead of arterial blood samples which represent the FDA's standard when it comes to evaluating new spO_2_ measuring devices [21]. Lastly, all studies used manually triggered measurements when the person was in an appropriate position at rest. Therefore, the performance of the Apple Watch when it does automatic measurements as a person is going about their day could not be assessed.

## Conclusions

Our review suggests that the Apple Watch Series 6 does not show a strong systematic bias compared to conventional, medical-grade pulse oximeters. However, outliers appear to occur fairly often even though we could not determine a definitive frequency and should not cause concern in otherwise healthy individuals. The impact of skin color on measurement accuracy should be investigated.
